# Property Improvements of Silica-Filled Styrene Butadiene Rubber/Butadiene Rubber Blend Incorporated with Fatty-Acid-Containing Palm Oil

**DOI:** 10.3390/polym15163429

**Published:** 2023-08-17

**Authors:** Siwarote Boonrasri, Parichat Thipchai, Pongdhorn Sae-Oui, Sarinthip Thanakkasaranee, Kittisak Jantanasakulwong, Pornchai Rachtanapun

**Affiliations:** 1 Faculty of Engineering and Agro-Industry, Maejo University, Chiang Mai 50290, Thailand; 2 Philosophy Program in Nanoscience and Nanotechnology (International Program/Interdisciplinary), Faculty of Science, Chiang Mai University, Chiang Mai 50200, Thailand; parichat_thi@cmu.ac.th; 3 MTEC, National Science and Technology Development Agency (NSTDA), Pathum Thani 12120, Thailand; pongdhor@mtec.or.th; 4 Division of Packaging Technology, School of Agro-Industry, Faculty of Agro-Industry, Chiang Mai University, Chiang Mai 50100, Thailand; sarinthip.t@cmu.ac.th (S.T.); jantanasakulwong.k@cmu.ac.th (K.J.); 5 Center of Excellence in Agro Bio-Circular-Green Industry (Agro BCG), Chiang Mai University, Chiang Mai 50100, Thailand; 6 Center of Excellence in Materials Science and Technology, Faculty of Science, Chiang Mai University, Chiang Mai 50200, Thailand

**Keywords:** lauric acid, oleic acid, fatty acid, palm oil, silica, rubber

## Abstract

Using vegetable oils as a plasticizer or processing aid in green rubber products is becoming popular due to environmental concerns. However, differences in vegetable oil processing result in varying amounts of low-molecular-weight (low-MW) free fatty acids (FFAs) in their composition, which range from 2% to 30%. This research investigated how the properties of silica-filled styrene butadiene rubber (SBR) and butadiene rubber (BR) blends were affected by the presence of FFAs in palm oil (PO). The rubber compounds containing a 70/30 SBR/BR blend, 30 phr of silica, and 2 phr of bis-(3-triethoxysilylpropyl) tetrasulfide (TESPT), and the vulcanizing agents were prepared and tested. The PO content was kept constant at 20 phr, while the number of FFAs, i.e., lauric acid (LA), palmitic acid (PA), and oleic acid (OA), in PO varied from 10–30%. The viscosity, dynamic mechanical properties, morphology, cure characteristics, and mechanical properties of the rubber blend were then measured. Regardless of the FFA types, increasing FFA content in PO decreased scorch time, cure time, minimum torque, and viscosity. As the FFA content increased, the torque difference and crosslink density also increased, which led to higher hardness, modulus, tensile strength, and abrasion resistance. The FFA types had a slight effect on the vulcanizate properties, even though LA showed slightly better mechanical properties than PA and OA. The results reveal that FFAs in PO not only improve processability but also function as a co-activator in silica-filled sulfur-vulcanized SBR/BR blend compounds.

## 1. Introduction

Styrene butadiene rubber (SBR) and butadiene rubber (BR) blends have low mechanical properties due to the lack of strain-induced crystallization at standard room temperature (23 ± 2 °C). Generally, silane-treated silica is used in SBR/BR compounds to improve tensile strength, abrasion resistance, skid resistance, rolling resistance, and wet traction for tire products [1]. However, the incorporation of silica into SBR/BR blends at high loading requires a suitable quantity of plasticizer to reduce compound viscosity for easier processing and to improve filler dispersion [2,3]. Mineral oil, particularly distillate aromatic extract (DAE) oil, has been widely used as a rubber process oil in the rubber industry for decades due to its good compatibility with many rubber types and low cost. However, DAE contains a high amount of polycyclic aromatic hydrocarbons (PAHs), which are considered highly carcinogenic and have recently been banned in Europe and some other countries. Vegetable oils have recently gained much attention from researchers around the globe and are becoming good alternatives to replace petroleum oils in the production of green tire tread compounds [4,5]. They are derived from renewable resources, PAH-free, and low-cost. The replacement of petroleum plasticizers with vegetable oils helps to alleviate the effects of global warming and improves many rubber properties [6,7,8]. Palm oil (PO), soybean oil (SO), rapeseed oil (RO), sunflower seed oil (SSO), and palm kernel oil (PKO) are the most widely used vegetable oils in the market because they are produced worldwide at a very large scale and thus have a reasonable price, providing great potential to be applied as a plasticizer for the rubber industry [4,9,10,11,12]. These five types of oils account for about 91% of the total vegetable oil production of 208.8 million metric tons in 2022/2023 [13]. Applications of various vegetable oils in the rubber industry have been extensively studied during the last few decades. It has been reported that PO could be used to replace paraffinic oil in ethylene propylene diene rubber (EPDM) without having negative effects on processability and mechanical properties [14]. In addition, the PO, Moringa oil (MO), and Niger oil (NO) exhibited higher thermal stability than light naphthenic oil, and, in the case of silica-filled natural rubber (NR), it provided superior processing properties over light naphthenic oil [3,15]. Abbas and coworkers [16] found that the presence of unsaturated free fatty acid (FFA) in crude PO resulted in improved plasticization and enhanced the rubber–carbon black interaction.

Depending on the production process, the quantity of FFAs in vegetable oils can be largely varied. It was previously reported that the amounts of FFAs were approximately 15% in jatropha oil [17] and about 13% in coconut oil [18]. When used as a cooking oil, the FFAs in PO have to be removed until their content is less than 1% [17]. The amount of FFAs in vegetable oils depends greatly on how they are collected, processed, stored, aged, and degraded [5,19,20]. A small amount of FFAs in vegetable oils also comes from the partial breakdown of vegetable oils during production and storage [5,21,22]. There are many types of FFAs in vegetable oils, both saturated and unsaturated, such as lauric acid (LA), myristic acid (MA), palmitic acid (PA), and stearic acid (SA) [23]. It is well known that fatty acids are widely used together with metal oxides, particularly zinc oxide (ZnO), to activate the sulfur vulcanization reaction in rubber compounds, known as vulcanization activators. Currently, the most commonly used fatty acid is SA [24]. Activators have been defined as substances that enhance the effectiveness of accelerators during sulfur vulcanization. At high temperatures, ZnO reacts with SA to form rubber-soluble zinc stearate and then facilitates the cross-linking reaction [25,26]. Jayewardhana et al. [11] reported that SO could be used as a processing aid and a co-activator in rubber compounding instead of petroleum-based aromatic oils. Generally, fatty acids are classified into four groups by the chain length or the number of carbon atoms in their aliphatic chain. Short-chain fatty acids have 5 or fewer carbon atoms (e.g., butyric acid); medium-chain fatty acids have 6 to 12 carbon atoms (e.g., lauric acid); long-chain fatty acids have 13 to 21 carbon atoms (e.g., palmitic acid and oleic acid); very-long-chain fatty acids have 22 or more carbon atoms (e.g., lignoceric acid) [27,28]. Compared to vegetable oils, which are triglycerides (the products of the reaction between glycerol and fatty acids), FFAs in vegetable oils have much smaller molecular sizes and lower molecular weights (MW), i.e., the MWs of LA, PA, and OA are about 200.32, 256.42, and 282.46 g/mol, respectively. Compared with PO, which has an MW of about 859.40 g/mol, the MWs of most FFAs are approximately 3–4 times lower.

Despite the presence of FFAs in vegetable oils, the effects of FFA type and content on the processability and mechanical properties of rubber have not been thoroughly studied and reported. Thus, the present work aimed to investigate the use of PO, which contained various contents of FFAs (LA, PA, and OA), as a plasticizer and co-activator in silica-filled rubber blends. The prepared PO was mixed with a 70:30 SBR/BR blend, 30 phr of precipitated silica, and the vulcanizing agents. The amount of PO was fixed at 20 phr, while its FFA content was varied to include 0%, 10%, 20%, and 30%. Various properties, including viscosity, dynamic mechanical properties, morphology, cure characteristics, and mechanical properties, were measured and reported.

## 2. Materials and Methods

### 2.1. Materials

**Figure 1:**
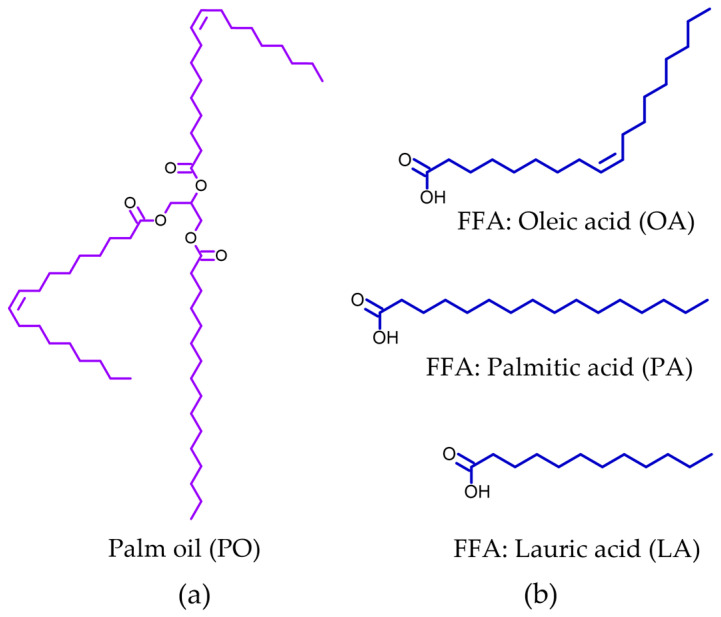
Chemical structure of (**a**) palm oil (PO) and (**b**) free fatty acids (FFAs) such as LA, PA, and OA.

The compounding ingredients and their suppliers used in this experiment are as follows: bis-(3-triethoxysilylpropyl) tetrasulfide (TESPT) from JJ Degussa Co., Ltd. (Bangkok, Thailand), precipitated silica (Tokusil-URT) with an average particle size of 30 nm and a BET specific surface area of 170 m^2^/g from Tokuyama Siam Silica Co., Ltd. (Rayong, Thailand), stearic acid and zinc oxide from Chemmin Co., Ltd. (Samuthprakarn, Thailand), sulfur from Siam Chemical Public Co., Ltd. (Bangkok, Thailand), 2-mercaptobenzothiazole (MBT) and tetramethylthiuram disulfide (TMTD) from Reliance Technochem Co., Ltd. (Bangkok, Thailand), palm oil (PO) from Morakot Industries PCL (Bangkok, Thailand), and all FFAs (LA, PA, and OA) from Union Science Co., Ltd. (Chiang Mai, Thailand). Figure 1 shows the chemical structures of PO, LA, PA, and OA. Various amounts of fatty acids (0%, 10%, 20%, and 30%) were added to palm oil. The mixture was stirred using a mechanical stirrer at 30 rpm for 30 min at room temperature prior to being used. Both styrene butadiene rubber (SBR 1502, with 23% styrene content) and butadiene rubber (BR 1220) were obtained from Lucky Four Co., Ltd. (Bangkok, Thailand).

### 2.2. Compound Preparation

The rubber compounds, based on the formulations given in Table 1, were prepared using a laboratory two-roll mill (Model YFTR-8, Yong Fong Machinery Co., Ltd., Samut Sakhon, Thailand). The mixing conditions were set as follows: roll gap of 0.5 mm and roll temperature of 60 °C. The rubbers were initially premixed on the two-roll mill for 2 min before adding silica, TESPT, and PO. The mixing was carried out for 8 min before adding stearic acid, ZnO, MBT, TMTD, and sulfur. After mixing for another 5 min, the rubber compounds were sheeted and stored at room temperature for 24 h before testing.

### 2.3. Morphology, Viscosity, and Dynamic Mechanical Properties

The silica particles and their dispersion in the rubber matrix were examined by a field emission scanning electron microscope (FE-SEM, model S-4700, Hitachi, Tokyo, Japan) at 3 kV electron energy. The new cryogenic-fractured surfaces of the rubber specimens were coated with Pt-Pd before the examination.

Mooney viscosity was measured using a Mooney viscometer (Prescott Instruments, Mooneyline MV, Tewkesbury, UK) at a test temperature of 100 °C following ISO 289-1 [29].

A capillary rheometer (Rheo-tester 2000, Göttfert, Germany) was used to measure the shear viscosity of the rubber compound. The rheometer was set at 100 °C and pre-heated for 180 s. The length-to-diameter ratio (L/D) of the die was 15:1.

A rubber process analyzer (MonTech, D-RPA 3000, Columbia, IN, USA) was used to evaluate the magnitude of filler–filler interaction by measuring the storage shear modulus (G′) of the rubber compounds under the strain-sweeping mode at 100 °C and 0.5 Hz. The difference in G′ at low (2%) and high (100%) strains (ΔG′), widely known as the “Payne effect,” was used to represent the degree of filler–filler interaction.

### 2.4. Cure Characteristics and Sample Preparation

The vulcanization of the silica-filled SBR/BR blend compound was determined using a moving die rheometer (MDR, model UR-2010, U-CAN Dynatex, Inc., Taichung, Taiwan) at 150 °C following ISO 6502-3 [30]. The rubber compounds were then vulcanized in a compression mold at 150 °C using an electrically heated hydraulic press (model HPC 100D, OOMN semi-automatic molding press, Shanghai Zimmerli Weili Rubber and Plastic Machinery Co., Ltd., Shanghai, China). For vulcanized sheets with 2 mm thickness, the optimum cure time (t_c_90) from the MDR was used during the curing process. For thicker specimens, a longer cure time was applied to compensate for the heat transfer effect. The vulcanized samples were stored at room temperature for at least 24 h before testing.

### 2.5. Mechanical Property Measurement

The hardness of the 6 mm thick specimen was measured at room temperature with a Shore A durometer (Digi Test, Bareiss, Stouffville, ON, Canada) according to ISO 48-4 [31]. The average value was reported from at least six readings taken from different positions.

A universal testing machine (Model 5566, Instron, Norwood, MA, USA) was used to measure the tensile properties of the rubber vulcanizates (2 mm thick) following ISO 37 [32]. The specimens (die type 1) were tested at 500 mm/min using a 1 kN load cell. The average of three measurements was reported.

The swelling test was performed by preparing the vulcanized specimens (3 cm × 1 cm × 2 mm), weighing them with an analytical balance, and immersing them in toluene. The specimens were left in toluene at room temperature and weighed regularly until they reached a constant weight.

The abrasion tester (Zwick model 6120, Zwick, Ennepetal, Germany) was utilized to measure the abrasion volume loss, which is inversely proportional to the abrasion resistance, of the rubber vulcanizates according to ISO 4649:2017 [33] (method A). The cylindrical specimens with a diameter of 16.0 ± 0.2 mm and a thickness of 12 mm were prepared and tested by abrading against an abrasive paper with 60-grade corundum at a speed of 0.32 m/s and a force of 10 N. Equation (1) was used to calculate the volume loss of the specimens after being abraded for 40 m. The average abrasion volume loss of five specimens was reported.

(1) $$A=\frac{{{∆m}_{t}\;\times \;S}_{0}}{d_{t}\;\times \;S}$$

where

A = abrasion loss (mm^3^),
∆m_t_ = mass loss of the test specimen (mg),
d_t_ = density of the test specimen (mg/mm^3^),
S_0_ = normal abrasiveness (200 mg),
S = abrasiveness of the abrasive paper (mg).

## 3. Results and Discussion 

### 3.1. Effect of FFAs on Morphology, Viscosity, and Dynamic Mechanical Properties

**Figure 2:**
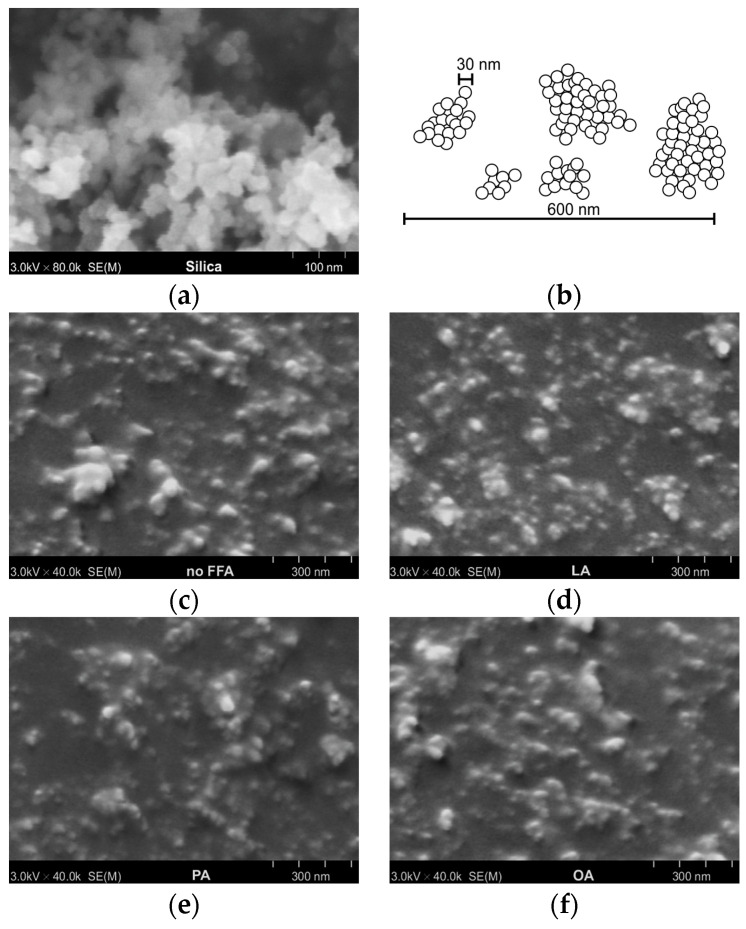
FE-SEM micrographs of (**a**) the silica powder, (**b**) a proposed schematic representation of silica aggregates, (**c**) the rubber blend plasticized with FFA-free PO, (**d**) the rubber blend plasticized with PO having 10% LA, (**e**) the rubber blend plasticized with PO having 10% PA, and (**f**) the rubber blend plasticized with PO having 10% OA.

Figure 2 shows FE-SEM micrographs of the silica powder and silica-filled rubbers containing PO with 10% of different FFA types. The average primary particle size of the silica powder (Figure 2a,b) was about 30 nm. The primary particles were not isolated but formed large clusters called agglomerates. When mixed with rubbers on the two-roll mill, the shearing forces caused the breakdown of silica agglomerates into aggregates, as evidenced by the smaller particle size of silica embedded in the rubbers, as shown in Figure 2c–f. The results reveal that FFA type had little effect on filler dispersion degree because all SEM micrographs of the rubber samples showed a comparable degree of dispersion with aggregate sizes ranging from 100 to 600 nm. Good filler dispersion was achieved because—in addition to the presence of a silane coupling agent, which can significantly reduce the filler–filler interaction—the ester group in PO and the carboxylic group in FFAs (if any) are both polar and can form H-bonds with silanol groups on the silica surfaces (the proposed model will be provided later, leading to a reduction in filler–filler interactions and improved filler dispersion [3,9,15]. This explanation is supported by Yan et al., who employed surface-selective solid-state nuclear magnetic resonance (ssNMR) to prove that the ester group of soybean oil can form H-bonds with the silanol group on silica surfaces [34]. In addition, Mensah et al. have recently used a wide-angle X-ray diffraction (WAXD) technique to show that PO could improve carbon black dispersion in both polar and non-polar rubbers [35].

**Figure 3:**
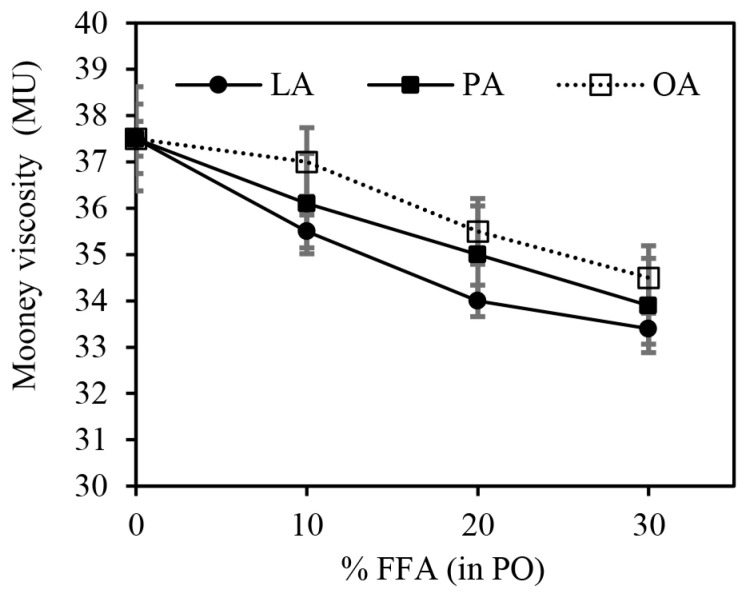
The effects of different types and contents of FFA in PO on the Mooney viscosity of the silica-filled SBR/BR blend compounds.

Figure 3 shows the Mooney viscosities of the silica-filled SBR/BR blend compounds with different types and contents of FFA. The Mooney viscosity tended to decrease as the FFA content increased, regardless of the FFA type. This is because of the greater plasticization effect of the low-MW FFA as compared to the high-MW PO. The results indicate that the presence of any type of FFA in PO facilitates the flowability of the rubber compounds and, thus, improves processability. A possible reason is given to the smaller molecular size of FFA, causing a reduction in PO viscosity and, hence, compound viscosity. When incorporated into rubbers, FFA, which is much smaller than PO, can get into the voids between rubber chains very easily, giving it a greater lubricating effect than PO. The rubber chain’s mobility is therefore enhanced with increasing FFA content in PO. As can be observed, LA showed the lowest compound viscosity at any given FFA content, followed by PA and OA, probably due to it having the lowest MW or molecular size. However, the difference was not significant, and, hence, it can be roughly said that the Mooney viscosity of the rubber compounds was not greatly affected by the FFA type.

**Figure 4:**
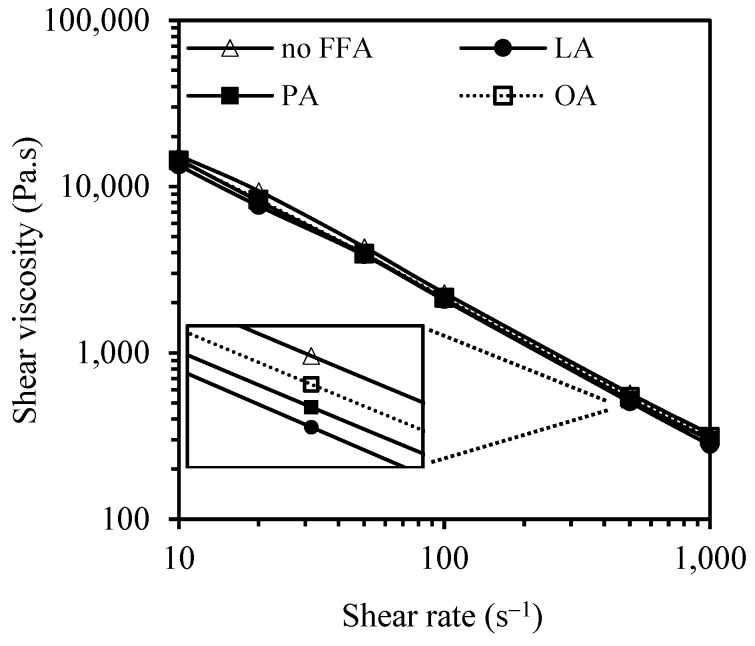
Dependence of shear viscosity on the shear rate of the rubber compounds.

Figure 4 shows the shear viscosity of the silica-filled SBR/BR blend compounds, plasticized with PO having different FFA types at 10%, as a function of the shear rate obtained from the capillary rheometer. As expected, shear viscosity decreased as the shear rate increased, indicating a shear thinning behavior. A closer look reveals that, at any given shear rate, the rubber compound plasticized with FFA-free PO showed a higher shear viscosity than the ones plasticized with 10% FFA. The results agree well with those from Mooney viscosity.

**Figure 5:**
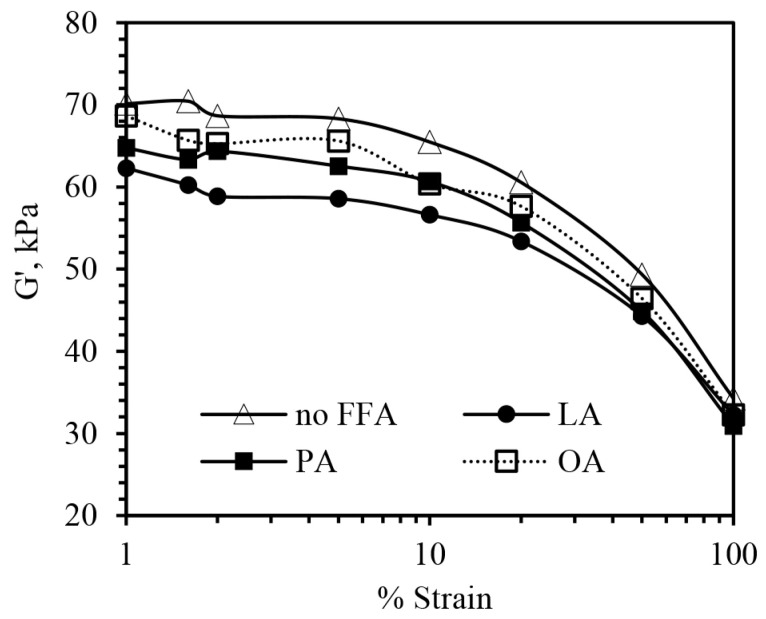
The effect of FFA type (at 10% PO) on storage modulus (G′) as a function of the strain of the silica-filled SBR/BR blend compounds.

Figure 5 shows the dynamic mechanical properties of the rubber compounds measured by RPA. The results indicate that G′ decreased with increasing strain for all compounds, which means that the silica network breaks down with increasing strain [3,36].

Generally, the difference between G′ values at low and high strains (ΔG′ or Payne effect) reflects the degree of filler–filler interaction (filler network) [3,36]. The results in Figure 5 and Table 2 show that the compound containing FFAs had a lower ΔG′ and a lower degree of filler–filler interaction, which is the consequence of the greater plasticization effect of FFAs, as previously mentioned. It is widely known that filler–filler interaction depends on filler type, filler dispersion level, plasticizer type, and plasticizer content. It has also previously been reported that, at a similar degree of filler dispersion, vegetable oil, which has the greatest plasticization effect and the lowest viscosity, provides the lowest level of filler–filler interaction [36].

**Figure 6:**
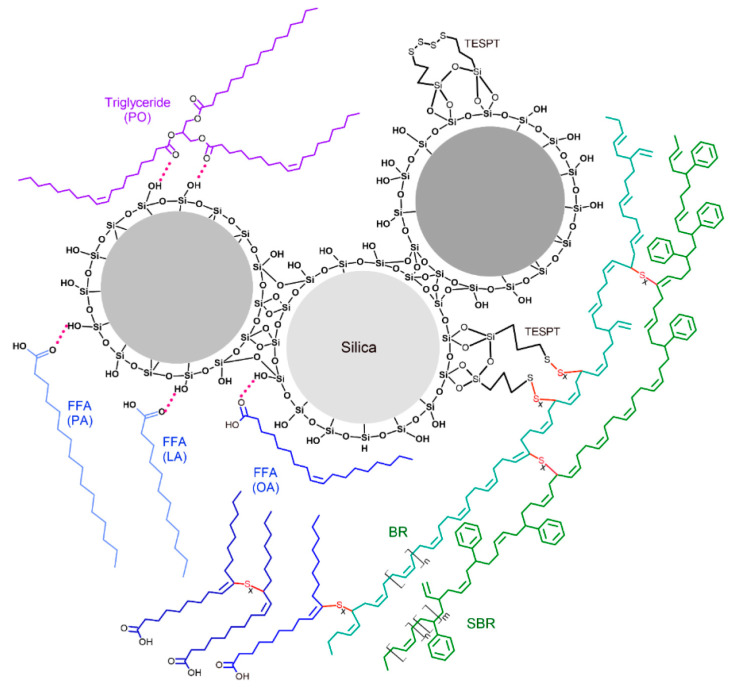
Proposed model of the H-bond formation between the silanol groups on the silica surface and PO or the crosslinks between OA and OA or OA and rubber molecules.

It is widely known that the addition of a silane coupling agent greatly improves rubber–filler interaction by forming covalent bonds to both rubbers and silica and, thus, facilitates filler dispersion by weakening the filler–filler interaction. In the presence of vegetable oils or FFAs, the polar ester groups of PA and carboxylic groups of FFAs (if any) can also form hydrogen bonds (H-bonds) with the silanol groups on the silica surfaces, as depicted in the proposed model shown in Figure 6. As OA contains double bonds in its molecules, there is the possibility that OA could form sulfidic linkages with the rubbers or other OA molecules.

### 3.2. Effect of FFAs on Cure Characteristics

**Figure 7:**
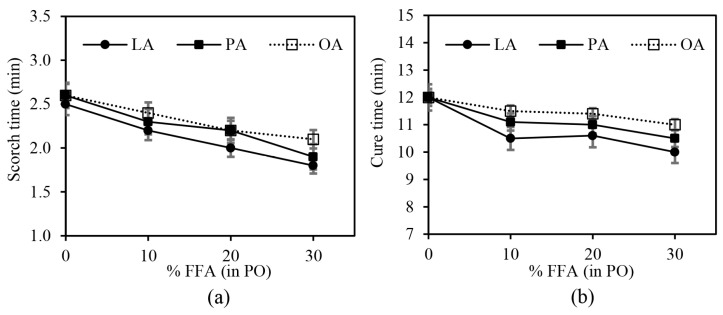
The effects of FFA type and content in PO on (**a**) scorch time and (**b**) cure time of the silica-filled SBR/BR blend compounds.

The effects of FFA type and content on the cure characteristics of the silica-filled SBR/BR blend compounds are illustrated in Figure 7a,b for scorch time (t_s2_) and optimum cure time (t_c_90). Both t_s2_ and t_c_90 tended to decrease slightly with increasing FFA content, regardless of the FFA type. This could be explained by the fact that FFAs have a lower MW and can form hydrogen bonds with the silanol groups on the silica surface more effectively than PO, thus providing better wetting on the silica surface. The reduction in silanol groups on the silica surface results in reduced activator adsorption, leading to a faster cure rate. In addition, the FFAs also function as an organic activator by reacting with inorganic ZnO to form rubber-soluble zinc soap or zinc carboxylate, which is the reactive intermediate for sulfur vulcanization [5]. At a given FFA content, LA showed a slightly shorter cure time than PA and OA, respectively. This observation is consistent with the previously published work, which reported that the vulcanization reaction was slower when the aliphatic chain length of fatty acids increased [26].

**Figure 8:**
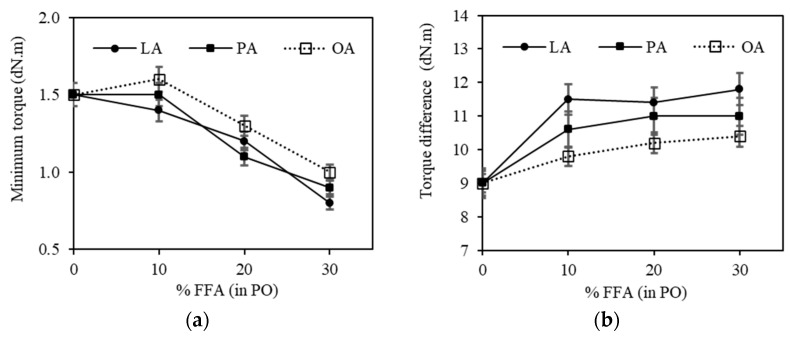
The effects of FFA type and content in PO on (**a**) minimum torque and (**b**) the torque difference of the silica-filled SBR/BR blend compounds.

Figure 8a shows the minimum torque (M_L_) of the silica-filled SBR/BR blend compounds containing PO with different FFA types and contents. The M_L_ decreased as the FFA content increased. This is attributed to the greater plasticization effect of FFA compared with PO, as previously mentioned. The results are in good agreement with the Mooney viscosity and shear viscosity results.

The torque difference (max. torque–min. torque) of the silica-filled SBR/BR blend compounds containing PO with different FFA types and contents is shown in Figure 8b. The results indicate that the FFA type affects the torque difference of the silica-filled SBR/BR blend compounds. LA had a slightly higher torque difference than PA and OA, possibly because of its small and saturated molecules. Moreover, at the same FFA content, the number of LA molecules in PO was the highest because it has the lowest MW. The FFA in PO will react with ZnO and help activate the vulcanization reaction, as mentioned earlier [5]. Similar findings have been found in silica-filled natural rubber (NR), in which the presence of FFA in PO increased the vulcanization rate, resulting in increased crosslink density [37]. The torque difference of OA (unsaturated fatty acid) was slightly lower than that of the other fatty acids. This is because OA contains double bonds, which could consume some sulfur atoms in the compound by reacting with the double bonds of the rubbers or other OA molecules as previously shown in Figure 6. The production of factice by vulcanizing unsaturated vegetable oils with sulfur at high temperatures is a good example to confirm that the double bonds in fatty acids or PO can be crosslinked and, hence, consume some sulfur atoms from the rubber matrix [5]. Other studies have reported that the crosslinking agent can react with the unsaturated fatty acids in soybean oil, which can reduce the crosslinking density and affect the mechanical properties [38,39].

### 3.3. Effect of FFAs on Physical Properties of the Vulcanizates

**Figure 9:**
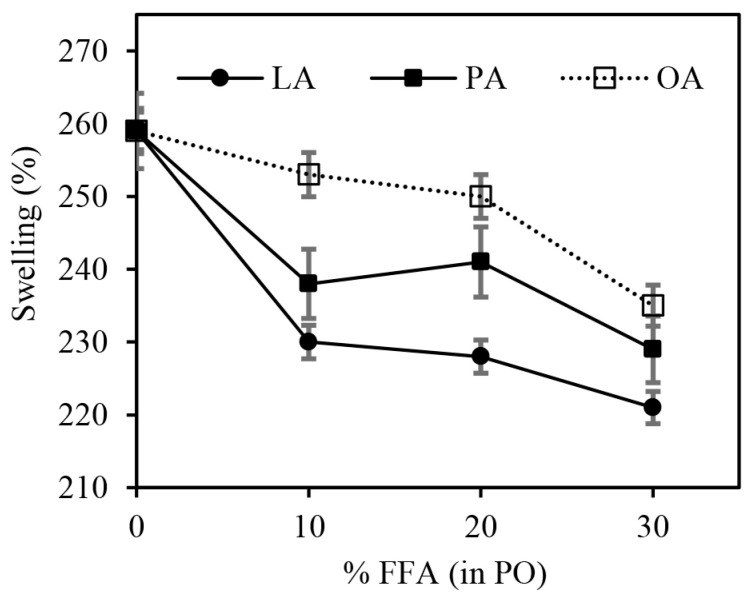
The effects of FFA type and content in PO on the swelling degree of the silica-filled SBR/BR blend compounds.

Figure 9 shows the swelling degree of the silica-filled SBR/BR blend vulcanizates. The swelling degree decreased as the FFA content increased, regardless of the FFA type. It is a fact that the swelling degree of the rubber vulcanizate generally decreases as the crosslink density increases [40,41]. The torque difference is therefore inversely related to the swelling degree, as shown in Figure 8b and Figure 9. The swelling results matched well with the torque difference results, i.e., LA had slightly higher crosslink density (lower swelling degree) than PA and OA when compared at the same FFA content. The same explanation applies. It should be noted that the migration of compounding ingredients, including PA and fatty acids, is unavoidable during the swelling test, and it is assumed that the differences in degree of chemical migration among all samples are negligible.

**Figure 10:**
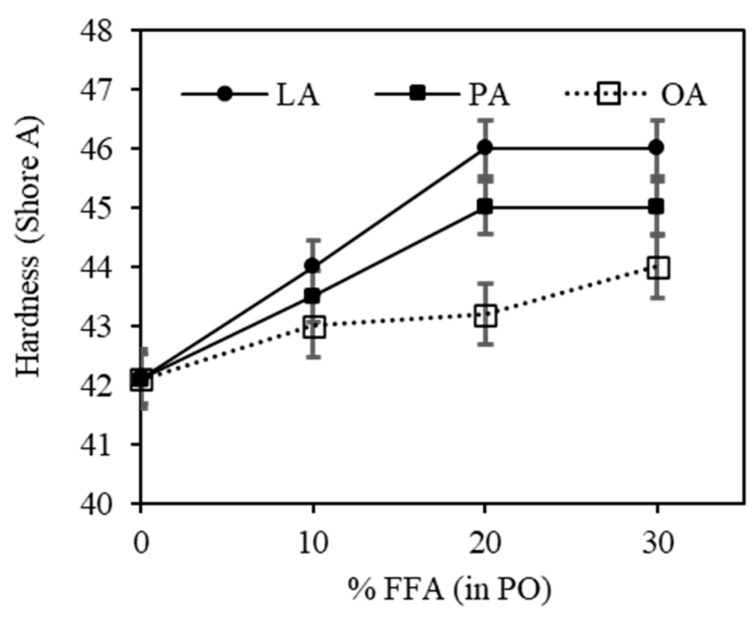
The effects of FFA type and content in PO on the hardness of the silica-filled SBR/BR blend compounds.

The hardness results of the rubber vulcanizates are displayed in Figure 10. The hardness increased gradually as the FFA content increased due to the increased crosslink density, as evidenced by the torque difference and swelling results. For LA and PA, the increase in hardness with FFA content was found up to 20% before leveling off. At any given FFA content, LA had the highest hardness, followed by PA and OA, respectively. Again, the explanation is given by its highest crosslink density, as previously mentioned.

**Figure 11:**
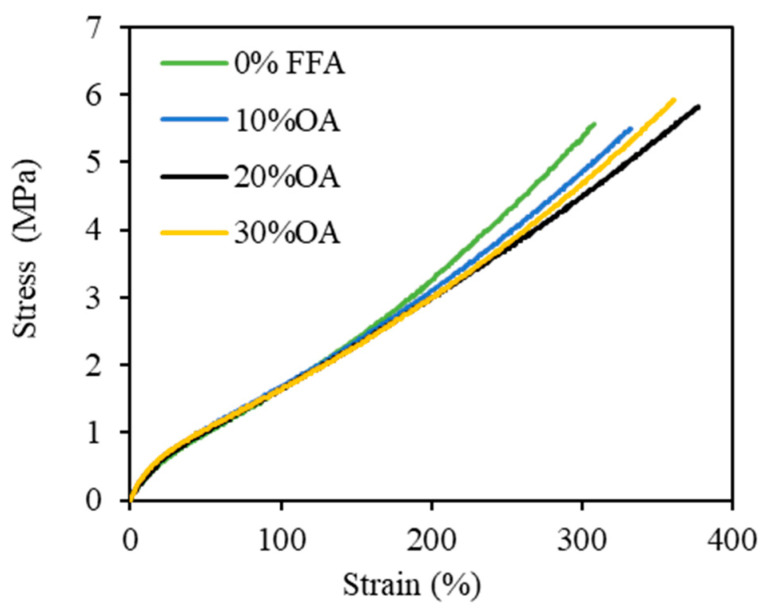
Examples of stress–strain curves of the rubber vulcanizates incorporated with PO containing various OA contents.

**Figure 12:**
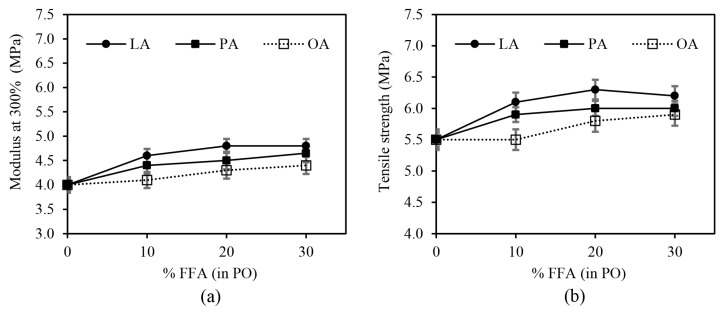
The effects of FFA type and content in PO on (**a**) 300% modulus and (**b**) tensile strength of the silica-filled SBR/BR blend compounds.

Figure 11 displays the examples of stress–strain curves of the rubber vulcanizates. Figure 12a,b show the 300% modulus and tensile strength of the silica-filled SBR/BR blend vulcanizates plasticized with PO having different FFA types and contents. The tensile properties were slightly affected by the FFA type, i.e., the LA system had slightly higher modulus and tensile strength than the other fatty acid systems. The modulus and tensile strength of the rubber vulcanizates also increased as the FFA content increased up to 20%. A further increase in FFA content showed no significant effect on the modulus and tensile strength of the vulcanizates, particularly in the LA and PA systems. The initial improvement in modulus and tensile strength is due to the improved crosslink density with increasing FFA content. However, at high FFA contents (>20%), the greater plasticization effect of FFA becomes more pronounced and overrides the increased crosslink density.

Compared with LA and PA, which are saturated fatty acids, OA showed the lowest modulus, hardness, and tensile strength. This is because OA contains double bonds, which can consume curatives in the rubber matrix, as previously mentioned. The tensile strength of the silica-filled SBR/BR blend vulcanizates tended to increase with FFA content up to a certain point before leveling off. Other researchers have reported that the factice formation by self-vulcanization of unsaturated fatty acids in vegetable oils could lead to a reduction in the tensile strength of rubber [16,42].

**Figure 13:**
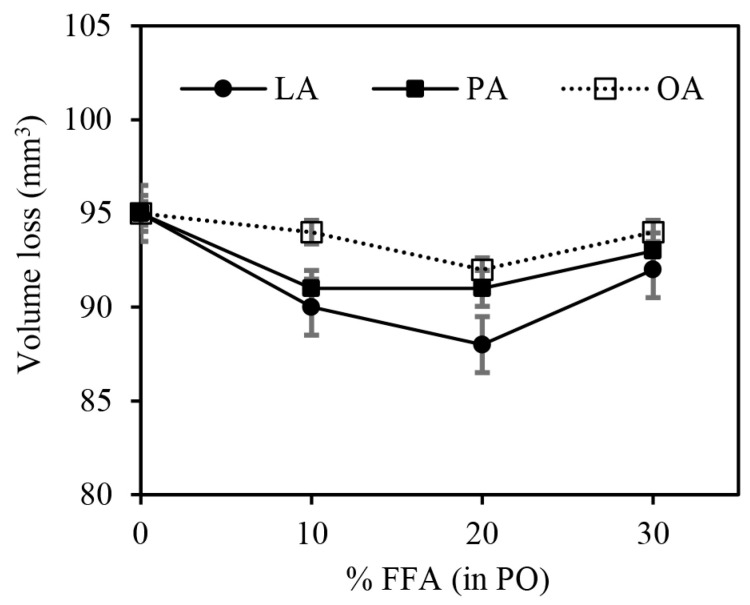
The effects of FFA type and content in PO on volume loss of the silica-filled SBR/BR blend compounds.

As shown in Figure 13, an increase in FFA content in PO slightly enhanced the abrasion resistance of the rubber vulcanizates up to approximately 20% of FFA in PO, as can be seen from the gradual decrease in the volume loss. The improvement in abrasion resistance is attributed to the increased crosslink density. However, at higher FFA content (>20%), impaired abrasion resistance was observed, probably due to the dominant effect of plasticization over the crosslink density. At any given FFA content, OA had the lowest abrasion resistance due to it having the lowest values of crosslink density and the possible formation of factice (crosslinked fatty acid or crosslinked vegetable oil) within the rubber matrix, which could ruin the abrasion resistance.

## 4. Conclusions

The findings of this study indicate that FFA content in PO greatly affects the processability and mechanical properties of the rubber vulcanizates because FFA can function not only as a good plasticizer but also as an organic activator for the sulfur vulcanization reaction. The increase in FFA content leads to improved processability, enhanced crosslink density, and, hence, mechanical properties such as hardness, modulus, and tensile strength. Among the FFAs used in this study, LA showed slightly better processability than the other FFAs due to it having the lowest MW (shortest chain length) and also provided the highest mechanical properties. On the other hand, OA showed the lowest processability and mechanical properties because it had double bonds that could consume sulfur from the rubber matrix, leading to a lower crosslink density when compared with PA and LA, as evidenced by the torque difference and swelling test results. This study explored the use of PO containing various types and contents of FFA as a plasticizer and co-activator in sulfur-vulcanized silica-filled SBR/BR blend compounds.

## Figures and Tables

**Table 1:** The compound formulations used in the present study.

Ingredients	Content, phr ^a^									
	0% FFA in PO	%LA in PO			%PA in PO			%OA in PO		
		10	20	30	10	20	30	10	20	30
SBR 1502	70.0	70.0	70.0	70.0	70.0	70.0	70.0	70.0	70.0	70.0
BR 1220	30.0	30.0	30.0	30.0	30.0	30.0	30.0	30.0	30.0	30.0
ZnO	5.0	5.0	5.0	5.0	5.0	5.0	5.0	5.0	5.0	5.0
Stearic acid	0.5	0.5	0.5	0.5	0.5	0.5	0.5	0.5	0.5	0.5
Silica	30.0	30.0	30.0	30.0	30.0	30.0	30.0	30.0	30.0	30.0
TESPT	2.0	2.0	2.0	2.0	2.0	2.0	2.0	2.0	2.0	2.0
MBT	1.0	1.0	1.0	1.0	1.0	1.0	1.0	1.0	1.0	1.0
TMTD	0.5	0.5	0.5	0.5	0.5	0.5	0.5	0.5	0.5	0.5
Sulfur	2.0	2.0	2.0	2.0	2.0	2.0	2.0	2.0	2.0	2.0
PO	20.0	18.0	16.0	14.0	18.0	16.0	14.0	18.0	16.0	14.0
LA	0.0	2.0	4.0	6.0	-	-	-	-	-	-
PA	-	-	-	-	2.0	4.0	6.0	-	-	-
OA	-	-	-	-	-	-	-	2.0	4.0	6.0

^a^ Parts per hundred rubber (phr).

**Table 2:** Payne effect of the rubber compounds incorporated with PO containing 10% FFAs.

	No FFA	LA	PA	OA
ΔG′ (kPa)	34.41	26.60	30.43	30.99

## Data Availability

The data presented in this study are available on request from the corresponding author.
